# Long noncoding RNA X-inactive-specific transcript promotes hepatic fibrosis by suppressing ferroptosis in hepatic stellate cells via the miR-663a/GPX4 axis

**DOI:** 10.3389/fphys.2025.1734886

**Published:** 2026-02-11

**Authors:** Jing Dai, Guo-Hui Zhong, Jun-Xing Yang, Xiao-Yu Tan, Dong Dai, Ming-Yi Li

**Affiliations:** 1 Department of Hepatobiliary and Pancreatic Surgery, Affiliated Hospital of Guangdong Medical University, Zhanjiang, Guangdong, China; 2 Department of Hepatobiliary and Pancreatic Surgery, Central People’s Hospital of Zhanjiang, Zhanjiang, Guangdong, China

**Keywords:** glutathione peroxidase 4, hepatic fibrosis, hepatic stellate cells, long noncoding RNA X-inactive-specific transcript, miR-663a

## Abstract

**Aim:**

Hepatic fibrosis (HF) is a critical pathological stage in the progression of chronic liver diseases, where hepatic stellate cell (HSC) activation is a key event. Ferroptosis regulates the fate of HSCs and represents a potential anti-fibrotic target. Long non-coding RNA XIST (lncRNA-XIST) is involved in fibrosis-related diseases. This study investigated how lncRNA-XIST promotes HF by regulating ferroptosis through the microRNA-663 (miR-663a)/GPX4 axis.

**Methods:**

LX-2 HSCs were activated using ethanol at varying concentrations for different durations to determine optimal conditions. HSCs were intervened with small interfering RNA against lncRNA-XIST, and Liproxstatin-1 was applied. RT-qPCR, Western blotting, CCK-8, colony formation, LDH release, and biochemical assays assessed gene/protein expression, cell viability, proliferation, ferroptosis markers (Fe^2+^, MDA, GSH), and cell death. Dual-luciferase assays validated interactions among lncRNA-XIST, miR-663a, and GPX4. *In vivo*, an HF mouse model was established and treated with sh-XIST or miR-663a antagonists. Liver fibrosis was evaluated by histology, immunohistochemistry, and serum liver injury markers (ALT, AST, HYP).

**Results:**

Ethanol promoted LX-2 activation and upregulated lncRNA-XIST in a time- and dose-dependent manner (optimal: 100 mM, 24 h). LncRNA-XIST knockdown reduced α-SMA, CoL1A1, GPX4 levels, and cell proliferation while increasing ferroptosis markers (indicative of enhanced ferroptosis) and miR-663a expression. Mechanistically, lncRNA-XIST was found to act as a competing endogenous RNA (ceRNA) to sponge miR-663a, thereby upregulating GPX4 and inhibiting ferroptosis. *In vivo*, lncRNA-XIST was shown to promote HF progression via the miR-663a/GPX4 axis.

**Conclusion:**

LncRNA-XIST promotes HF by acting as a ceRNA for miR-663a, regulating GPX4, and suppressing ferroptosis to activate HSCs.

## Introduction

Hepatic fibrosis (HF) is a dynamic and tightly regulated molecular, cellular, and tissue process that is driven by a diverse population of hepatic myofibroblasts, and leads to the excessive deposition of extracellular matrix components (Parola and Pinzani, 2019). Activation of hepatic stellate cells (HSCs) facilitates extracellular matrix, protease and pro-inflammatory cytokine secretion, which further triggers cellular fibrosis (Trivedi et al., 2021). HSCs represent the primary cellular origin of myofibroblasts that secrete matrix proteins, playing a pivotal role in HF (Higashi et al., 2017). HF stands as one of the prominent contributors of both mortality and morbidity on a global scale, but unfortunately the availability of efficacious pharmaceutical interventions remains limited (Ellis and Mann, 2012). Hence, to enhance patients’ survival rates, there is a pressing necessity to enhance comprehension of the initiation and advancement of HF. Furthermore, more studies are imperative to pinpoint novel biomarkers and develop new therapeutic targets.

Ferroptosis, an emerging form of programmed cell death, is distinguished by an excess of intracellular iron and the buildup of lipid reactive oxygen species (Yuan et al., 2022). Ferroptosis is typically characterized by the impairment of the lipid repair enzyme, glutathione peroxidase 4 (GPX4), significant iron accumulation, and the peroxidation of polyunsaturated fatty acids (Pan et al., 2021). Ginsenoside Rg3 inhibits the methylation of ACSL4 through microRNA (miR)-6945-3p-mediated DNMT3B inhibition, consequently enhancing HSC ferroptosis and alleviating HF (Hu et al., 2024). Ferroptosis is identified as a crucial form of cell death in the progression of HF (Huang et al., 2022). Currently, specific anti-fibrotic drugs for HF are still lacking. Recently, ferroptosis induction has been proposed as a new strategy to eliminate activated HSCs (Zhu et al., 2025). Therefore, in-depth analysis of the regulatory mechanisms of ferroptosis in HF holds significant theoretical and clinical importance.

Long non-coding RNAs (lncRNAs) are a class of non-coding RNA molecules longer than 200 nucleotides in length, which are implicated in the modulation of gene expression through various mechanisms, such as cis/trans regulation, interactions with competitive endogenous RNAs (ceRNAs), and protein interactions (Chen, 2016). Notably, lncRNAs play a vital regulatory role in HF (Bian et al., 2019). For instance, lncRNA NEAT1 has been identified as a suppressor of HF in alcoholic steatohepatitis mice through the promotion of miR-129-5p and the inhibition of SOCS2, ultimately slowing down the development of alcoholic steatohepatitis (Ye et al., 2020). Also, lncRNA Airn is beneficial to HF by preserving the differentiation of liver sinusoidal endothelial cells (Chen et al., 2022). Of note, lncRNA X-inactive-specific transcript (XIST) subdues ferroptosis and stimulates lung adenocarcinoma growth by manipulating GPX4 (Lu et al., 2023). Limited research has been conducted to explore the potential of lncRNA-XIST in modulating GPX4 to regulate ferroptosis and its impact on HF.

miRNAs are short endogenously expressed RNAs that are capable of modulating the expression levels of various RNA molecules (Kilikevicius et al., 2022). The competitive endogenous RNA (ceRNA) hypothesis, which elucidates the interplay among various RNA molecules, delineates the modulation of gene expression by non-coding RNAs, such as lncRNAs, through competitive binding to miRNAs (Tay et al., 2014). LncRNA-XIST potentially exerts a significant regulatory function in metabolic and immune-related processes involved in liver regeneration via a ceRNA mechanism (Dai et al., 2023). LncRNA-XIST expression is increased in fibrotic liver tissues and may be a potential target for HF treatment; it may function to mediate mitochondrial dysfunction in hepatocytes via the miR-539-3p/ADAMTS5 axis (Wu et al., 2023). It has been recently documented that several lncRNAs can regulate GPX4 expression to affect ferroptosis through various mechanisms, such as the ceRNA mechanism (He et al., 2021) and activation of upstream transcription factors of GPX4 (Li et al., 2024). Herein, our bioinformatics analysis dependent on Starbase and TargetScan databases suggested miR-663a as a possible downstream target miRNA of lncRNA-XIST, with target sites that binds to GPX4. A recent study has reported that miR-663a suppresses the proliferation and activation of HSCs, thereby curbing HF (Guo et al., 2020). However, whether lncRNA-XIST regulates GPX4 expression via miR-663a to inhibit ferroptosis in HSCs and promote their activation, thereby promoting HF development, requires further investigation. The objective of this study was to explore the potential role of the lncRNA-XIST in modulating cellular ferroptosis to facilitate HF progression by modulating miR-663a and its downstream target protein, GPX4.

## Materials and methods

### Cell culture

Human hepatocellular carcinoma cell lines (HepG2, Huh7, and PLC/PRF/5) purchased from ATCC (Manassas, VA, United States) were cultured in the Dulbecco’s modified Eagle medium supplemented with 10% fetal bovine serum, penicillin (100 IU/mL), and streptomycin (100 mg/mL) [all obtained from Gibco (Grand Island, NY, United States)] and placed in an incubator at 37 °C with 5% CO_2_ and 95% humidity.

HSCs LX-2 cells (FH0108, Fuheng Biology, Shanghai, China) were cultivated in LX-2 complete medium (FH-LX-2, Fuheng Biology) at 37 °C, with 5% CO_2_ in the air and a humidity of 95% (Xie et al., 2019).

### Dose/Time-response study of ethanol and the ferroptosis inhibitor Liproxstatin-1 (Li)

1. LX-2 cells were treated with 0 mM, 50 mM, 100 mM and 150 mM ethanol for 24 h. Changes in cell viability were assessed via the CCK-8 assay. The expression levels of α-smooth muscle actin (α-SMA) and collagen type I alpha 1 chain (CoL1A1) were determined using Western blot.
2. LX-2 cells were treated with 100 mM ethanol for 0 h, 12 h, 24 h and 48 h. CCK-8 assay was conducted to assess cell viability, and α-SMA and CoL1A1 levels were determined by Western blot. Changes in RNA expression levels of lncRNA-XIST, miR-663a and GPX4 were measured using RT-qPCR assay.
3. After ethanol treatment, the activated LX-2 cells were transfected with small interfering RNA against lncRNA-XIST (si-XIST) and treated with 0 μM, 0.5 μM, 1 μM, and 2 μM Li, respectively, for 48 h. Cell viability was evaluated via CCK-8 assay, and level changes in ferroptosis markers, Fe^2+^, malondialdehyde (MDA) and glutathione (GSH), were detected by the kits.

### Cell line transfection and grouping

si-XIST and miR-663a inhibitors and their corresponding negative controls (NCs) (GenePharma, Shanghai, China) were introduced into LX-2 cells using Lipofectamine®2000 (Invitrogen, Carlsbad, CA, United States). All operations were performed as per the guidelines. Cells were gathered after 48-h transfection for subsequent experiments. The sequences were as follows: si-XIST: sense: 5′-GCAAAUGAAAGCUACCAAU-3’; antisense: 5′-AUUGGUAGCUUUCAUUUGC-3’; the miR-663a inhibitor: 5′-UCCGCCCCGCGGCGCCCUGGCG-3’. Sequences of si-NC and inhi-NC were nonsense RNA sequences with no homology to known gene sequences.

LX-2 cells were grouped as follows (detailed in Supplementary Table 1): (1) the Con group (cultured normally); (2) the LX-2 group (induced with ethanol for 24 h); (3) the LX-2 + si-NC group (induced with ethanol for 24 h and then transfected with si-NC for 48 h); (4) the LX-2 + si-XIST group (24 h ethanol induction and then 48 h transfection with si-XIST) (Xie et al., 2019); (5) the LX-2 + si-XIST + Li group [24 h ethanol induction, 48 h transfection with si-XIST, followed by treatment with 1 μM Li (a ferroptosis inhibitor; SML1414, Sigma-Aldrich, St Louis, MO, United States) for 48 h (Li D. et al., 2023)]; (6) the LX-2 + si-XIST + Vehicle group [24 h ethanol induction, 48 h transfection with si-XIST, followed by treatment with the same amount of solvent as Li (50% PEG300 and 50% saline) for 48 h]; (7) the LX-2 + si-XIST + inhi-NC group (induced with ethanol for 24 h and then co-transfected with si-XIST and 10 nM inhibitor NC for 48 h); (8) the LX-2 + si-XIST + miR-663a inhi group (induced with ethanol for 24 h and then co-transfected with si-XIST and 10 nM miR-663a inhibitor for 48 h). Each experiment was independently repeated 3 times.

### Reverse transcription-quantitative polymerase chain reaction (RT-qPCR)

Total RNA was extracted using TRIzol reagent (Invitrogen) and then reverse transcribed into complementary DNA using the PrimeScript RT reagent kit (Takara, Dalian, Liaoning, China). TaqMan primers and probes for the detection were all obtained from Takara. qPCR was carried out on the ABI PRISM 7900 sequence detection system of SYBR Green II (Takara). The program of PCR consisted of pre-denaturation at 95 °C for 5 min, and 40 cycles of denaturation at 95 °C for 15 s, annealing at 60 °C for 20 s, and extension at 72 °C for 35 s. With β-actin utilized as an internal reference gene for lncRNA-XIST and GPX4, and U6 as an internal reference gene for miR-663, respectively, data were normalized using the 2^−ΔΔCt^ method (Livak and Schmittgen, 2001). The primer sequences are listed in Table 1. All primers were synthesized by Sangon Biotechnology (Shanghai, China).

**TABLE 1:** Primer sequences.

Gene	Forward primer (5′-3′)	Reverse primer (5′-3′)
LncRNA-XIST	TAGTCCTCTGCGGCTTCC	TGCTGATCGTTTGGTGCT
GPX4	GCCGTGTAGATATGGTACAAGGA	CTGCTCTTCCAGAGGTCCTG
β-actin	CATGTACGTTGCTATCCAGGC	CTCCTTAATGTCACGCACGAT
miR-663a	GTGCGTGTCGTGGAGTCG	TTTAGGCGGGGCG
U6	ATTGGAACGATACAGAGAAGATT	GGAACGCTTCACGAATTTC

### Cell counting kit (CCK)-8 assay

LX-2 cells were seeded onto 96-well plates at a density of 5 × 10^3^ cells per well. The cell viability of each group at 0, 24, 48 and 96 h was evaluated using the CCK-8 kit (CA1210, Solarbio, Beijing, China). Briefly, cells were added with 100 μL/well CCK-8 working solution and then incubated at 37 °C for 2 h. The optical density (OD) at 450 nm was assessed using a microplate reader (Thermo Fisher Scientific, Waltham, MA, United States).

### Colony formation assay

LX-2 cells were seeded onto 6-well plates (500 cells per well) and resuspended for 2 weeks. Upon the visible formation of colonies to the unaided eye, the 6-well plates were taken. After the removal of the culture medium, cells were fixed with 2 mL/well methanol for 30 min, followed by the discarding of the methanol. Afterwards, cells were stained with 2 mL/well of 0.1% crystal violet (548-62-9, MACKLIN, Shanghai, China) for 3 min, and then observed and counted using a microscope (Olympus, Tokyo, Japan).

### Immunofluorescence assay

Lipid peroxidation (LPO) levels in LX-2 cells were detected using the BODIPY 581/591 C11 fluorescent probe (HY-D1301, MCE, Monmouth Junction, NJ, United States). Briefly, LX-2 cell coverslips were prepared and treated with 5 μM BODIPY 581/591 C11 working solution to ensure the coverage of all cells. Next, cell coverslips were fostered for 30 min at room temperature in the dark, washed with phosphate buffered saline, and sealed with an anti-fluorescence quencher. Finally, cells were observed and photographed under a fluorescence microscope (Olympus). The BODIPY 581/591 C11, in the absence of LPO, exhibited orange-red fluorescence, whereas the presence of LPO resulted in the manifestation of green fluorescence in BODIPY 581/591 C11. The LPO level in cells could be evaluated by analyzing the intensity of green fluorescence/red fluorescence.

### Establishment of the HF mouse model

A total of 42 male C57BL/6 mice (6–8 weeks old, weighing 20 ± 2 g, Guangdong Shuanglin Biopharmaceutical Co., Ltd., Zhanjiang, Guangdong, China) were maintained in a specific pathogen-free facility under standard conditions of 24 °C ± 2 °C, 50% ± 10% humidity, and a 12 h light-dark cycle.

After adaptive feeding for 1 week, mice were injected intraperitoneally with 10 mL/kg olive oil (#56–23-5, MACKLIN) containing 10% carbon tetrachloride (CCl_4_) twice a week for 8 consecutive weeks to induce HF, thereby establishing an HF mouse model (Yuan et al., 2022). Olive oil was utilized as a solvent for CCl_4_, and the solvent control group received an intraperitoneal injection of an equivalent volume of olive oil.

A total of 42 mice were arranged into 7 groups (six mice per group) as below: (1) the Normal group (normal control mice without any treatment); (2) the Vehicle control group (solvent control group; mice injected with equivalent volume of olive oil intraperitoneally); (3) the Model group (HF mice); (4) the short hairpin (sh)-NC and sh-XIST groups [sh-NC or sh-XIST lentivirus (pGLVU6/GFP vector; GenePharma) was dissolved in 0.5 mL of saline at a titer of 1 × 10^9^ TU/mL, and then injected into HF mice via tail vein once a week for 4 consecutive weeks] (Xie et al., 2019); (5) the sh-XIST + anta NC and sh-XIST + anta miR groups [equal amounts of sh-XIST and antagonist NC or antagonist miR-663a (5 μg per mouse) were dissolved in 1.5 mL saline, and then injected into HF mice via tail vein once a week for 4 consecutive weeks (Chai et al., 2020)].

The day after intervention, mice were weighed and 0.3 mL blood was taken from the orbit for enzyme-linked immunosorbent assay (ELISA). Next, liver tissues were collected, photographed, and weighed after sacrifice of mice with 150 mg/kg of 0.5% pentobarbital sodium (P3761, Sigma-Aldrich). The liver weight ratio (liver weight/weight × 100%) was calculated and normalized with the Normal group. There were six mice in each group. The right lobe of the liver of each mouse was utilized for immunohistochemistry, hematoxylin-eosin (H&E) staining and Masson staining, and the left lobe of the liver for Western blot and kit assays.

### Histopathological examination

H&E staining and Masson staining were applied for histopathological examination of mouse liver tissues. Liver tissue samples were immersed in 10% formalin solution, dehydrated in gradient ethanol, embedded in paraffin, and subsequently sectioned into slices with a thickness of 4 μm. The paraffin sections were routinely dewaxed and hydrated, and later subjected to staining with the H&E staining kit (60524ES60, Yeasen Company, Shanghai, China) and Masson staining kit (60532ES58, Yeasen Company), respectively. The experiment was conducted in strict accordance with the kit instructions. The samples were then observed and photographed under an optical microscope (Olympus). Collagen deposition in mouse liver tissues stained with Masson’s trichrome was quantified and analyzed using Image Pro Plus 6.0 (Media Cybernetics, Silver Spring, MD, United States). Collagen deposition volume fraction (CVF) = collagen fiber area/total area of liver tissue sections * 100%. There were six mice in each group. Three discontinuous sections were obtained from each mouse, and five non-overlapping fields of view were randomly selected from each section for statistical analysis. The results were expressed as the mean value.

### Alanine aminotransferase (ALT), aspartate aminotransferase (AST), and hydroxyproline (HYP) assay

Serum levels of ALT, AST and HYP, as well as HYP levels in liver tissue supernatant, were determined using ALT kit (C009-2-1, JianCheng Bioengineering Institute, Nanjing, Jiangsu, China), AST kit (C010-2-1, JianCheng Bioengineering Institute) and HYP kit (A030-2-1, JianCheng Bioengineering Institute). The specific experimental procedures were conducted following the manufacturer’s instructions.

### Western blot

The collected tissues and LX-2 cells were added with lysis buffer (AR0107, Boster, Wuhan, Hubei, China) to extract proteins. After the determination of concentration using the bicinchoninic acid kit (AR1189, Boster), protein samples were added with an appropriate amount of loading buffer and heated in a boiling water bath for 5 min for protein denaturation. The denatured protein samples were added onto the loading wells and separated by sodium dodecyl sulfate polyacrylamide gel electrophoresis. Subsequently, samples were electrotransferred onto the polyvinylidene fluoride membranes, which were then blocked in 3% bovine serum albumin (AR0184, Boster) for 2 h, and later cultivated with the following primary antibodies: anti-α-SMA (1:1000, #19245, CST, Beverly, MA, United States), anti-CoL1A1 (1:1000, #39952, CST) and anti-GPX4 (1:1000, ab125066, Abcam, Cambridge, United Kingdom) overnight at 4 °C, with glyceraldehyde-3-phosphate dehydrogenase (1:2500, ab9485, Abcam) as an internal reference. Thereafter, membranes were incubated with goat anti-rabbit immunoglobulin G (IgG) H&L [horseradish peroxidase (HRP)] (1:2000, ab6721, Abcam) at room temperature in the dark for 1 h, followed by development with enhanced chemiluminescence working solution (AR1191, Boster). Grayscale quantification of each group of bands in Western blot images was performed using Image Pro Plus 6.0 (Media Cybernetics). Glyceraldehyde-3-phosphate dehydrogenase (GAPDH) served as an internal reference protein, and the results were depicted as the ratio of relative GAPDH. The experiment was conducted thrice.

### Determinations of Fe^2+^, MDA, and GSH

Fe^2+^, MDA, and GSH levels were assayed utilizing the Fe detection kit (ab83366, Abcam), MDA detection kit (ab118970, Abcam), and reduced GSH detection kit (BC1175, Solarbio), respectively. Specific experimental procedures were conducted in light of the manufacturer’s manuals.

### Lactate dehydrogenase (LDH) assay

Cytotoxicity was quantified using the LDH kit (A020-2-2, Jiancheng Bioengineering Institute). After cells were centrifuged at 600 g and 4 °C for 5 min, the supernatant was collected. The kit working solution was added into the 96-well plates sequentially. Later, cells were incubated at 37 °C for 30 min, with the OD value measured at 450 nm. The specific experimental steps were carried out according to the instructions.

### Bioinformatics analysis

The binding sites between lncRNA-XIST and miR-663a were predicted using the Starbase database (https://rnasysu.com/encori/), and the binding sites between miR-663a and GPX4 were predicted utilizing the TargetScan database (http://www.targetscan.org/vert_72/).

### Dual-luciferase reporter gene assay

Wild-type (WT) and mutant (MUT) plasmids were synthesized and inserted into the lncRNA-XIST 3′-untranslated region (3′UTR) (lncRNA-XIST-WT/MUT) or GPX4 3′UTR (GPX4-WT/MUT) using the pMIR-reporter vector (KL-ZL-1015-01, Ke Lei Biological Technology, Shanghai, China), respectively. LX-2 cells in the logarithmic phase were seeded onto 96-well plates. Upon reaching 70% cell confluence, cells were co-transfected with 100 ng of lncRNA-XIST WT and MUT plasmids (lncRNA-XIST-WT/MUT) or GPX4 WT and MUT plasmids (GPX4-WT/MUT), along with mimics NC or miR-663a mimics. Following a 48 h transfection period, the relative activity of luciferase was evaluated using the luciferase detection kit (HY K1013, MCE) to evaluate whether miR-663a had a targeted binding relationship with the 3′UTR of lncRNA-XIST or GPX4, respectively.

### Immunohistochemistry

Liver tissues were paraffin-embedded and repaired under high temperature and high pressure for 2 min, followed by blockade with goat serum (SL038, Solarbio) for 20 min at room temperature. After overnight incubation with anti-α-SMA (1:500, #19245, CST) or anti-CoL1A1 (1:500, #39952, CST) at 4 °C, the sections were incubated with goat anti-rabbit antibody IgG H&L (HRP) (1:1000, ab214050, Abcam) for 60 min at 37 °C. Subsequently, the sections were subjected to diaminobenzidine staining (DA1016, Solarbio), hematoxylin staining (H8070, Solarbio), and photography under a microscope (Olympus). Quantitative analysis of immunohistochemistry was conducted using Image Pro Plus 6.0 (Media Cybernetics).

### Statistical analysis

Data were statistically analyzed and graphed using GraphPad Prism 8.01 (GraphPad Software, San Diego, CA, United States). Six mice were assigned into each group in animal experiments (n = 6), and cellular experiments were repeated thrice independently (n = 3). Normal distribution of continuous variables was tested using the Kolmogorov-Smirnov test. Normally distributed data were expressed as mean ± standard deviation (SD). Data comparisons between two groups were conducted using an independent sample *t*-test; data comparisons among multiple groups were performed using one-way analysis of variance (ANOVA), followed by Tukey’s multiple comparisons test for post-hoc analysis. Statistical significance was defined as *P* < 0.05. The actual difference between the two groups of data was evaluated dependent on the effect size (Cohen’s d). The formula was as follows: Cohen‘s d = (mean difference between the two groups)/(combined standard deviation of the two groups), with d > 0.8 regarded as a large effect size, which is indicative of an extremely significant difference between the two groups of data.

## Results

### Ethanol induced lncRNA-XIST expression in HSCs, and knockdown of lncRNA-XIST promoted ferroptosis to suppress HSC activation

**FIGURE 1:**
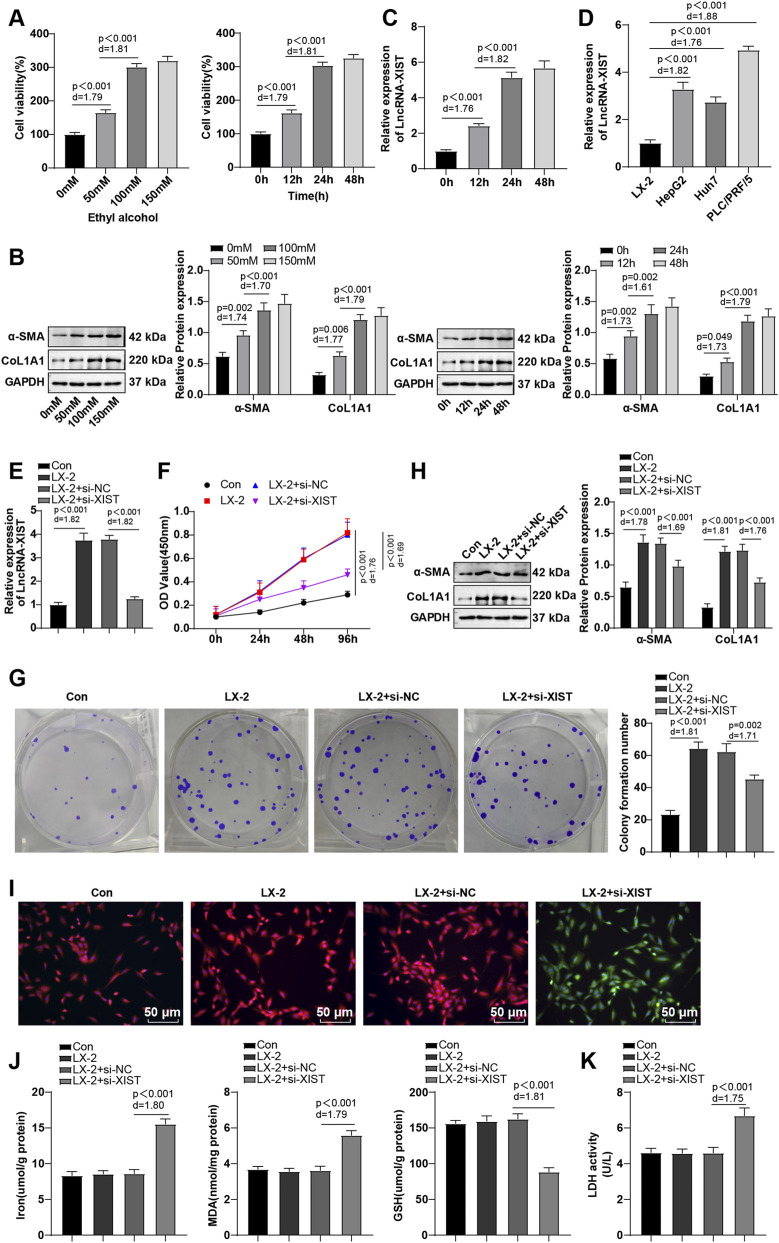
Ethanol induced lncRNA-XIST expression, and silencing of lncRNA-XIST promoted ferroptosis to repress HSC activation. LX-2 cells were treated with 0 mM, 50 mM, 100 mM, and 150 mM ethanol for 24 h or with 100 mM ethanol for 0 h, 12 h, 24 h, and 48 h, respectively, to induce HSC activation. **(A)** CCK-8 assay to evaluate cell viability; **(B)** Western blot to determine α-SMA and CoL1A1 expression; **(C)** RT-qPCR to determine lncRNA-XIST expression; **(D)** RT-qPCR to measure the lncRNA-XIST level in activated LX-2 cells and hepatocellular carcinoma cells (HepG2, Huh7, and PLC/PRF/5). LX-2 cells were treated with 100 mM ethanol for 24 h, and **(E)** RT-qPCR to determine lncRNA-XIST mRNA expression; **(F)** CCK-8 to evaluate cell viability; **(G)** Colony formation assay to assess cell proliferation; **(H)** Western blot to measure α-SMA and CoL1A1 levels; **(I)** BODIPY 581/591 C11 immunofluorescence to observe LPO levels (for cells without LPO, the BODIPY 581/591 C11 fluorescent probe showed orange-red fluorescence; for cells with LPO, the BODIPY 581/591 C11 fluorescent probe exhibited green fluorescence); **(J)** Kits to determine Fe^2+^, MDA and GSH levels; **(K)** LDH assay to assess cell death. The cellular experiments were repeated three times independently and the data were depicted as mean ± SD. Data comparisons among multiple groups were performed by one-way ANOVA, with *post hoc* analysis using Tukey’s multiple comparison test. Differences were considered statistically significant at *P* < 0.05. The actual difference between the two groups of data was assessed using the effect size Cohen’s d (d) (LX-2 group *vs*. Con group; LX-2 + si-XIST group *vs*. LX-2 + si-NC group), with d > 0.8 considered a large effect size.

LX-2 cells were treated with 0 mM, 50 mM, 100 mM, and 150 mM ethanol for 24 h or with 100 mM ethanol for 0 h, 12 h, 24 h, and 48 h, respectively, to induce HSC activation. CCK-8 assay results illustrated that ethanol enhanced the cell viability of LX-2 cells dose- and time-dependently, and the LX-2 cell viability reached its peak and was stable upon ethanol treatment at a concentration of 100 mM for 24 h (Figure 1A, all *P* < 0.001). The expression levels of HSC activation markers α-SMA and CoL1A1 (Xie et al., 2019) were further examined. It was found that ethanol promoted α-SMA and CoL1A1 expression dose- and time-dependently, and α-SMA and CoL1A1 levels were the highest and remained stable under ethanol treatment at 100 mM for 24 h (Figure 1B, all *P* < 0.05). The above results suggested that 100 mM and 24 h were the optimal concentration and time of ethanol treatment, respectively. As reflected by RT-qPCR results, lncRNA-XIST expression increased with the increase of ethanol treatment time and remained stable at 24-48 h (Figure 1C, all *P* < 0.001). In addition, the differential expression of lncRNA-XIST in activated LX-2 cells and several hepatocellular carcinoma cell lines that might be linked with fibrosis (HepG2, Huh7, and PLC/PRF/5) was analyzed, with the results showing that the lncRNA-XIST level in hepatocellular carcinoma cells was further upregulated (Figure 1D, all *P* < 0.001). These results revealed that lncRNA-XIST might increase with HF progression.

Subsequently, LX-2 cells were treated with 100 mM ethanol for 24 h to induce their activation according to the experimental conditions determined in the pre-experiment. Colony formation assay further revealed that ethanol evidently increased cell proliferation (*P* < 0.001) (Figure 1G). Moreover, after transfection with si-XIST, LX-2 cells displayed decreased lncRNA-XIST mRNA level. The corresponding alterations in cellular function further suggested effective transfection, as evidenced by reduced cell viability and α-SMA and CoL1A1 expression levels (*P* < 0.05), and diminished cell proliferation (*P* < 0.001) (Figures 1E–H). It is documented that promotion of ferroptosis represses HSC activation and mitigates HF (Kuo et al., 2020). The LPO assay with BODIPY 581/591 C11 fluorescent probe found no prominent change in the ratio of orange-red and green fluorescence in the LX-2 cells after ethanol treatment, indicating that the cells did not exhibit substantial LPO (Figure 1I). Additional results revealed no significant changes in Fe^2+^, MDA, and GSH levels in cells after ethanol treatment (all *P* > 0.001) (Figure 1J). After transfection with si-XIST, the green fluorescence in LX-2 cells was intensified, indicating the occurrence of significant LPO (Figure 1I). Additionally, Fe^2+^ and MDA levels were increased (*P* < 0.01), and GSH level was reduced (*P* < 0.001) (Figure 1J). LDH assay results illustrated no substantial change in cell death after ethanol induction (*P* > 0.05), which was increased after transfection with si-XIST (*P* < 0.001) (Figure 1K). The results collectively indicate that ethanol induces lncRNA-XIST expression, and knockdown of lncRNA-XIST promotes ferroptosis to inhibit HSC activation.

### Suppression of ferroptosis partially nullified the effect of lncRNA-XIST knockdown on the activation of HSCs

**FIGURE 2:**
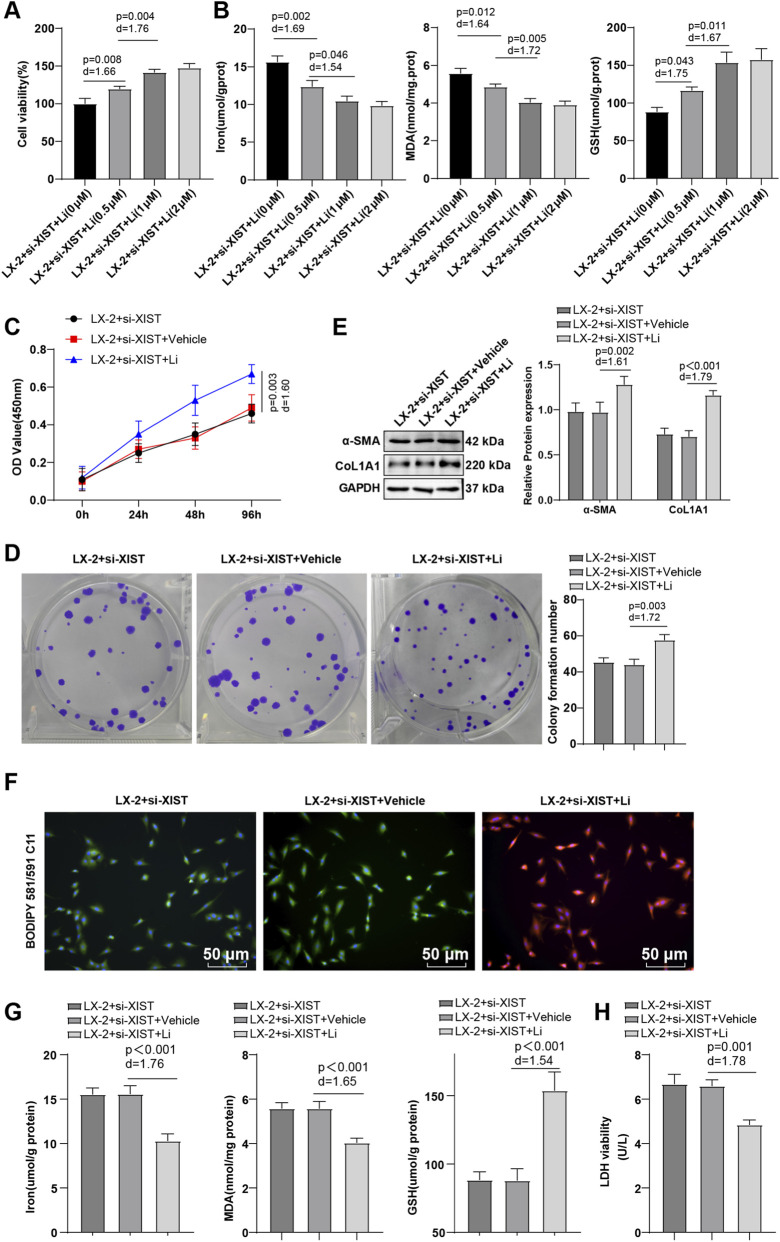
Inhibition of ferroptosis partially nullified the influence of lncRNA-XIST silencing on HSC activation. Activated LX-2 cells transfected with si-XIST were treated with 0, 0.5, 1, and 2 μM Li for 48 h. **(A)** CCK-8 to evaluate cell viability; **(B)** Kit detection to determine Fe^2+^, MDA, and GSH levels. Activated LX-2 cells transfected with si-XIST were treated with 1 μM Li for 48 h, and **(C)** CCK-8 to assess cell viability; **(D)** Colony formation assay to evaluate cell proliferation; **(E)** Western blot to determine α-SMA and CoL1A1 expression levels; **(F)** BODIPY 581/591 C11 immunofluorescence to observe LPO levels (for cells without LPO, the BODIPY 581/591 C11 fluorescent probe showed orange-red fluorescence; for cells with LPO, the BODIPY 581/591 C11 fluorescent probe exhibited green fluorescence); **(G)** Kit detection to determine Fe^2+^, MDA, and GSH levels; **(H)** LDH assay to assess cell death. Cell experiments were repeated three times independently, with data presented as mean ± SD. One-way ANOVA was applied for data comparisons among multiple groups, with *post hoc* analysis using Tukey’s multiple comparison test. Differences were considered statistically significant at *P* < 0.05. The actual difference between the two groups of data was assessed using the effect size Cohen’s d (d) (LX-2 + si-XIST + Li group *vs*. LX-2 + si-XIST + Vehicle group), with d > 0.8 considered a large effect size.

LX-2 cells were induced using ethanol for 24 h and then transfected with si-XIST while being treated with 0, 0.5, 1, and 2 μM Li for 48 h. CCK-8 assay indicated that Li dose-dependently restored LX-2 cell viability, with the Li treatment at 1 μM yielding the highest and stable cell viability (Figure 2A, all *P* < 0.05). Li dose-dependently downregulated Fe^2+^ and MDA levels and upregulated the GSH level, with the 1 μM concentration indicating the most pronounced effects (Figure 2B, all *P* < 0.05).

Subsequently, LX-2 cells transfected with si-XIST were treated with 1 μM Li. Cells in the LX-2 + si-XIST + Li group showed elevated LX-2 cell viability and α-SMA and CoL1A1 expression levels (*P* < 0.05), enhanced proliferation (*P* < 0.05), diminished green fluorescence, decreased Fe^2+^ and MDA levels (*P* < 0.05), raised GSH level (*P* < 0.05), and reduced cell death (*P* < 0.05) (Figures 2C–H). The aforementioned results suggest that inhibition of ferroptosis partially abrogates the effect of lncRNA-XIST knockdown on HSC activation.

### LncRNA-XIST acted as a ceRNA for miR-663a to regulate GPX4 expression

**FIGURE 3:**
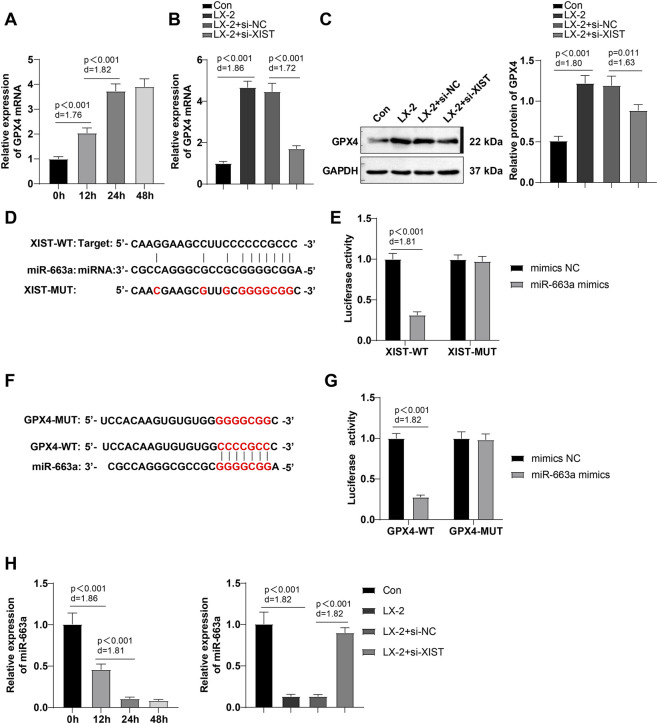
LncRNA-XIST functioned as a ceRNA for miR-663a to regulate GPX4 expression. **(A,B,H)** RT-qPCR to determine miR-663a and GPX4 mRNA expression levels; **(C)** Western blot to measure GPX4 level; **(D)** Starbase database to predict the target binding sites of lncRNA-XIST with miR-663a; **(E)** Dual-luciferase reporter gene assay to verify the binding relationship of lncRNA-XIST with miR-663a; **(F)** TargetScan database to predict the target binding site of miR-663a with GPX4; **(G)** Dual-luciferase reporter gene assay to verify the binding relationship between miR-663a and GPX4. The cellular experiments were conducted in triplicate independently and the data were expressed as mean ± SD. Comparisons of data among multiple groups were performed by one-way ANOVA, with *post hoc* analysis using Tukey’s multiple comparison test. Differences were considered statistically significant at *P* < 0.05. The actual difference between the two groups of data was assessed using the effect size Cohen’s d (d) (LX-2 group *vs*. Con group; LX-2 + si-XIST group *vs*. LX-2 + si-NC group), with d > 0.8 considered a large effect size.

LX-2 cells were treated with 100 mM ethanol for 0 h, 12 h, 24 h and 48 h. Changes in mRNA expression of GPX4 were detected by RT-qPCR, and it was observed that GPX4 mRNA was increased with the increase of ethanol treatment time, which tended to be stable at 24-48 h (Figure 3A, all *P* < 0.001). RT-qPCR and Western blot were conducted to determine GPX4 expression in different groups, with the results displaying that ethanol treatment dramatically raised GPX4 expression in cells (*P* < 0.001) (Figures 3B,C), which was reduced by lncRNA-XIST silencing (*P* < 0.01) (Figures 3B,C). Starbase database analysis manifested that miR-663a was a downstream target miRNA of lncRNA-XIST, and TargetScan database prediction showed that miR-663a had target sites binding to GPX4 (Figures 3D,F). Furthermore, dual-luciferase reporter gene assay verified the presence of the binding relations of miR-663a with lncRNA-XIST and GPX4, respectively (Figures 3E,G). RT-qPCR data revealed that miR-663a levels reduced with the increment of ethanol treatment time and stabilized at 24–48 h. Furthermore, ethanol decreased miR-663a level in cells, whereas silencing of lncRNA-XIST partly restored miR-663a level (*P* < 0.01) (Figure 3H). These results indicate that lncRNA-XIST acts as a ceRNA for miR-663a to regulate GPX4 expression.

### LncRNA-XIST promoted HSC activation largely through a miR-663a/GPX4-dependent suppression of ferroptosis

**FIGURE 4:**
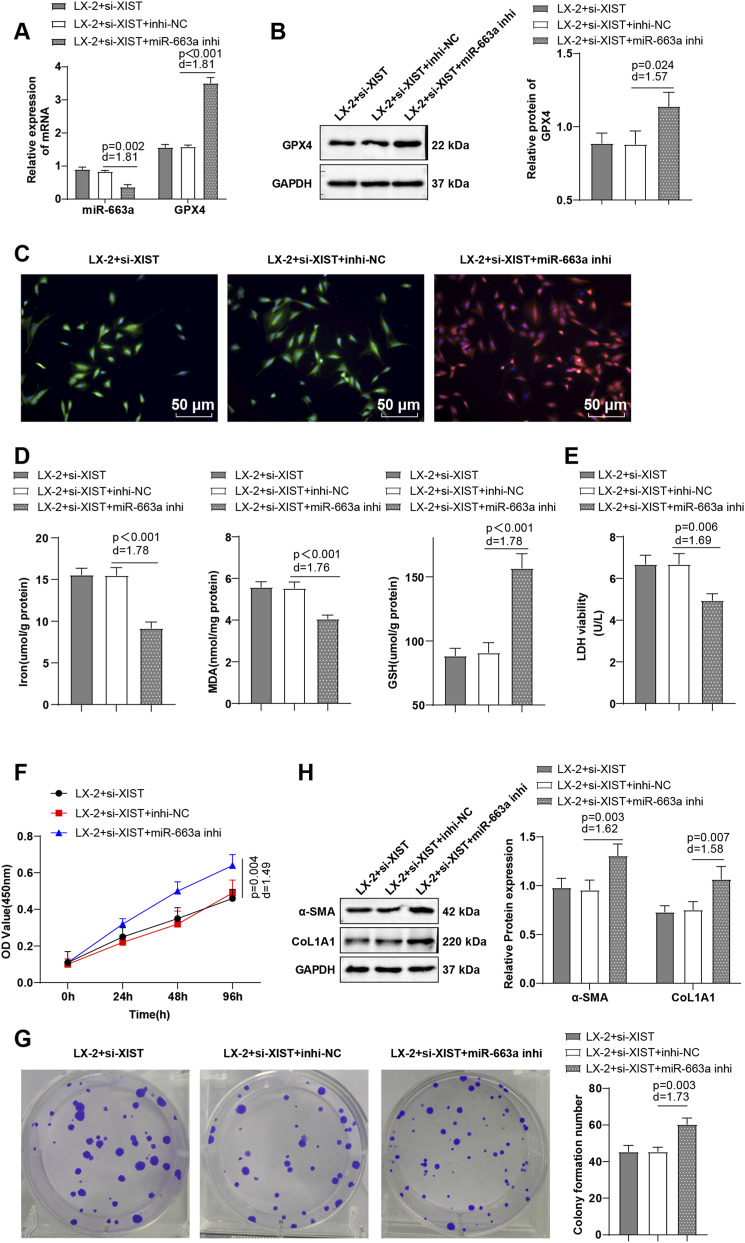
LncRNA-XIST promoted HSC activation via a miR-663a/GPX4-dependent suppression of ferroptosis. **(A)** RT-qPCR to determine miR-663a and GPX4 mRNA expression levels; **(B)** Western blot to determine GPX4 level; **(C)** BODIPY 581/591 C11 immunofluorescence to observe LPO levels (for cells without LPO, the BODIPY 581/591 C11 fluorescent probe showed orange-red fluorescence; for cells with LPO, the BODIPY 581/591 C11 fluorescent probe exhibited green fluorescence); **(D)** Kits to measure Fe^2+^, MDA, and GSH expression levels; **(E)** LDH assay to assess cell death; **(F)** CCK-8 to evaluate cell viability; **(G)** Colony formation assay to assess cell proliferation; **(H)** Western blot to measure α-SMA and CoL1A1 levels. The cellular experiments were repeated three times independently. Data were expressed as mean ± SD, and one-way ANOVA was utilized for data comparisons among multiple groups, with *post hoc* analysis using Tukey’s multiple comparison test. Differences were considered statistically significant at *P* < 0.05. The actual difference between the two groups of data was assessed using the effect size Cohen’s d (d) (LX-2 + si-XIST + miR-663a inhi group *vs*. LX-2 + si-XIST + inhi-NC group), with d > 0.8 considered a large effect size.

Cells in the LX-2 + si-XIST + miR-663a inhi group delineated decreased miR-663a level (*P* < 0.01), raised GPX4 level (*P* < 0.001), repressed LPO, reduced Fe^2+^ and MDA levels (*P* < 0.001), elevated GSH level (*P* < 0.001), diminished cell death (*P* < 0.01), increased cell viability and α-SMA and CoL1A1 expression levels (*P* < 0.01), and enhanced cell proliferation (*P* < 0.01) (Figures 4A–H). These data indicate that lncRNA-XIST promotes HSC activation largely through a miR-663a/GPX4-dependent suppression of ferroptosis.

#### LncRNA-XIST facilitated HF in mice by regulating miR-663a/GPX4

**FIGURE 5:**
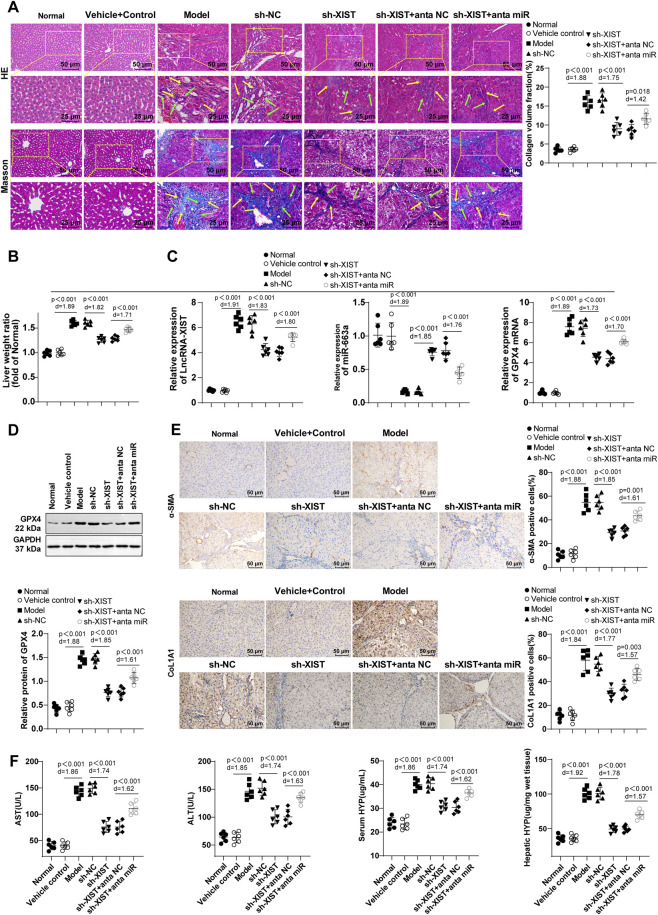
LncRNA-XIST promoted HF in mice by modulating miR-663a/GPX4. **(A)** H&E staining and Masson staining. The green arrows represent the inflammatory cell infiltration area, and the yellow arrows represent fibrotic scar and collagen deposition areas; **(B)** Mouse liver weight ratio; **(C)** RT-qPCR to measure lncRNA-XIST, miR-663a, and GPX4 mRNA expression levels in liver tissues; **(D)** Western blot to determine GPX4 expression; **(E)** Immunohistochemistry to determine α-SMA and CoL1A1 levels in liver tissues; **(F)** Kits to determine serum levels of ALT, AST, and HYP as well as HYP level in the liver tissue. n = 6. All experiments were independently repeated 3 times, and the data were expressed as mean ± SD. Data comparisons among multiple groups were conducted using one-way ANOVA, with *post hoc* analysis using Tukey’s multiple comparison test. Differences were considered statistically significant at *P* < 0.05. The actual difference between the two groups of data was assessed using the effect size Cohen’s d (d) (Model group *vs*. Vehicle control group; sh-XIST group *vs*. sh-NC group; sh-XIST + anta-miR group *vs*. sh-XIST + anta-NC group), with d > 0.8 considered a large effect size.

After CCl_4_ treatment, hepatocyte degeneration, inflammatory cell infiltration, fibrous scar and collagen deposition were all aggravated (Figure 5A), liver weight ratio was elevated (*P* < 0.01) (Figure 5B), lncRNA-XIST and GPX4 levels were boosted (*P* < 0.01), miR-663a level was decreased (*P* < 0.01) (Figures 5C,D), α-SMA and CoL1A1 levels in liver tissues were raised (*P* < 0.001) (Figure 5E), and serum levels of ALT, AST, and HYP as well as hepatic HYP levels were elevated (*P* < 0.001) (Figure 5F). Conversely, HF mice treated with sh-XIST showed reduced hepatocellular degeneration, inflammatory cell infiltration, fibrous scar and collagen deposition, and liver weight ratio (*P* < 0.001), reduced lncRNA-XIST and GPX4 levels (*P* < 0.001), elevated miR-663a level (*P* < 0.001), diminished α-SMA and CoL1A1 levels in liver tissues (*P* < 0.001), reduced serum ALT, AST, and HYP levels, and decreased hepatic HYP level (*P* < 0.001) (Figures 5A–F). Treatment with sh-XIST and antagonist miR-663a resulted in aggravations in hepatocyte degeneration, inflammatory cell infiltration, and fibrous scar and collagen deposition, an increase in mouse liver weight ratio (*P* < 0.001), elevations in lncRNA-XIST and GPX4 levels (*P* < 0. 001), a decrement in miR-663a level (*P* < 0.001), elevations in α-SMA and CoL1A1 levels in the liver tissues (*P* < 0.001), increments in serum ALT, AST, and HYP levels, as well as an elevation in the hepatic HYP level (*P* < 0.001) (Figures 5A–F). The aforementioned results indicate that lncRNA-XIST can promote HF in mice via miR-663a/GPX4.

## Discussion

**FIGURE 6:**
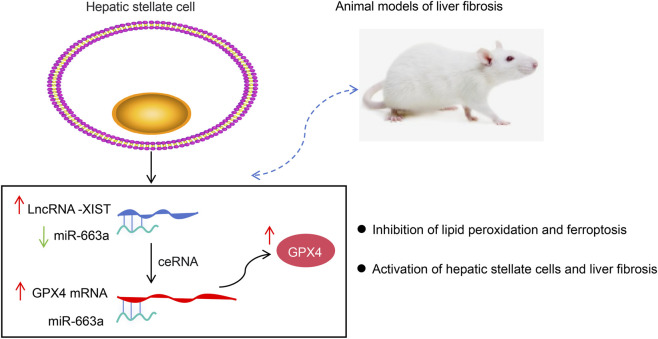
LncRNA_SIST acts as a competitive endogenous RNA(ceRNA) of miR-663a to regulate the expression of GPX4 to inhibit ferroptosis and peomote the activation of HSCs to promote liver fibrosis in mice.

HF resulting from chronic liver diseases of viral or metabolic etiology poses a significant challenge to global health (Roehlen et al., 2020). Pathogenetically, metabolic, toxic, or viral diseases result in hepatocyte damage and immune cell infiltration, triggering the trans-differentiation of HSCs into collagen-producing myofibroblasts (Elpek, 2014). Despite notable progress in comprehending the development of HF, the therapeutic efficacy of drugs employed in its treatment remains inadequate (Taymouri and Taheri, 2016). Therefore, it is crucial to identify efficient strategies for managing HF. Herein, the main innovations are as follows: 1) this study first indicates that ethanol induces lncRNA-XIST expression in HSCs and that lncRNA-XIST knockdown inhibits HSC activation by promoting ferroptosis; 2) lncRNA-XIST, as a ceRNA, relieves its suppression on the target gene GPX4 by adsorbing miR-663a, thereby maintaining GPX4 expression, suppressing ferroptosis, and ultimately promoting HSC activation (Figure 6); 3) this study further suggest that lncRNA-XIST inhibits ferroptosis and promotes HSC activation via the miR-663a/GPX4 axis, thereby contributing to the pathogenesis of HF in a mouse model.

HSCs represent the primary progenitor cells of myofibroblasts and play a crucial role in the fibrogenic response (Mederacke et al., 2013). In the normal liver, HSCs are non-proliferative perisinusoidal, quiescent cells, characterized by their high number of cytoplasmic lipid droplets and star-like morphology (Testerink et al., 2012). Upon liver injury, HSCs undergo activation and transition from a dormant state to a proliferative and contractile myofibroblast phenotype (Affo et al., 2017). LncRNA-XIST expression is raised in fibrotic liver tissues, suggesting its potential as a therapeutic target for HF treatment (Wu et al., 2023). The hard-done work of our peers has highlighted that lncRNA-XIST is elevated in LX-2 cells stimulated with ethanol, and knockdown of lncRNA-XIST reduces cell viability and diminishes protein levels of α-SMA and CoL1A1 (Xie et al., 2019). This study systematically screened and determined 100 mM ethanol treatment for 24 h as the optimal conditions for HSC activation through ethanol dose-time gradient experiments. This condition effectively induced the HSC activation phenotype, providing an experimental basis for establishing an *in vitro* HSC activation model. Our study similarly observed that ethanol triggered an increase in lncRNA-XIST expression in HSCs, and the suppression of lncRNA-XIST hindered HSC activation.

Moreover, ferroptosis has been shown to potentially enhance the development of HF (Yu et al., 2020). Knockdown of lncRNA-XIST substantially reduces lung adenocarcinoma cell viability and enhances ferroptosis, as evidenced by notable increases in reactive oxygen species, MDA, and Fe^2+^ levels (Lu et al., 2023). Intriguingly, our study unveiled that downregulation of lncRNA-XIST promoted ferroptosis, as indicated by elevated Fe^2+^ and MDA levels in cells, decreased GSH levels, and increased cell death. Ferroptosis plays a role in HSC activation and the advancement of HF (Cho et al., 2022). Inhibition of ferroptosis leads to upregulation of four HSC activation markers, including CoL1A1, α-SMA, desmin, and fibronectin, reduction of Fe^2+^ and MDA content, and enhancement of GSH and cell viability (Kong et al., 2019). It has been documented that PRDM1 can promote ferroptosis in HSCs by inhibiting GPX4 expression and that GPX4 overexpression increases ROS and MDA levels while decreasing expression of GPX4 protein and HSC activation markers, directly revealing the regulatory role of GPX4-dependent ferroptosis in HF (Wu W. et al., 2024). A recent study has proposed that GPX4 dysfunction can directly trigger ferroptotic liver injury in an animal model, strongly supporting GPX4 as a core regulator of hepatic ferroptosis (Ma et al., 2025). Another study conducted by Ma et al. also states that HIF-1α and GPX4 are key regulatory nodes of ferroptosis in Alzheimer’s disease, suggesting the common significance of ferroptosis pathways in various organ fibrotic or degenerative diseases (Khan et al., 2025). This evidence, from both the perspective of HF mechanisms and the cross-disease ferroptosis regulatory network, provides strong support for the selection of relevant markers such as ROS, MDA, and GPX4 as core ferroptosis indicators in this study.

Recent reports have proposed that lncRNA-XIST may function as a ceRNA for miRNAs (Zhang et al., 2019). Our findings suggested that lncRNA-XIST acted as a ceRNA for miR-663a to regulate GPX4 expression. Compelling studies have indicated the association of miRNAs with liver pathophysiology, specifically in relation to HSC activation and fibrosis development. For example, miR-146a, miR-29b, and miR-200a may have been identified as potential regulators that can influence the proliferation and activation of HSCs by targeting TGF-β1 (He et al., 2012; Sun et al., 2014). miR-663a mitigates HSC activation, accompanied by abated α-SMA and COL1A2 expression levels and suppressed proliferation, which were counteracted by treatment with a miR-663a inhibitor in TGF-β1-treated HSCs (Guo et al., 2020). The inhibition of HF by miR-663a has been evidenced through the suppression of the proliferation and activation of HSCs (Dong et al., 2023). Reportedly, lncRNA LINC00657 acts as a sponge for miR-590-3p to upregulate HIF-1α expression via the ceRNA mechanism, thereby promoting angiogenesis under oxidative stress (Bao et al., 2018). This finding provides important experimental support for the hypothesis in our study that lncRNA-XIST acts as a molecular sponge for miR-663a to reinforce the existence of similar ceRNA mechanisms in endothelial cells under oxidative stress.

Multiple recent studies conjointly revealed that, although fibrosis exhibits organ specificity, its regulatory networks often share core pathways and effector mechanisms. For instance, Xanthohumol alleviates cardiac fibrosis via the PTEN/AKT/mTOR pathway (Sun et al., 2021). Botrychium ternatum can reduce TGF-β1 and hydroxyproline expression in lung tissues, thereby exerting anti-pulmonary fibrotic effects (Lou et al., 2024). These findings highlight the common role of pathways such as TGF-β and AKT/mTOR in multi-organ fibrosis. Under this background, this study focuses on the regulation of ferroptosis by the lncRNA-XIST/miR-663a/GPX4 axis in HF, so as to explore a novel mechanism distinct from classical signaling pathways, thereby enriching the cross-organ regulatory landscape in fibrotic diseases. Notably, a recent exploration performed by Yu et al. revealed that NEK6 promoted the ubiquitin–proteasomal degradation of FOXN3 through phosphorylation at S412 and S416 sites, thereby relieving its inhibitory effect on Smad, ultimately driving pulmonary fibrosis progression (Yu et al., 2025). This mechanism underscores the critical role of post-translational modifications in fibrosis and, from a regulatory perspective, supports the rationale of this study in focusing on post-transcriptional regulation mediated by non-coding RNAs. Meanwhile, M2 macrophage-derived exosomal lncRNA AK083884 has been found to regulate metabolic reprogramming via the PKM2/HIF-1α axis, alleviating viral myocarditis (Zhang et al., 2024). Gong et al. revealed the key function of cuproptosis-related lncRNAs in oral cancer (Gong et al., 2024). Together, the two studies indicate that lncRNAs can exert diverse roles in different diseases by modulating metabolic reprogramming and novel forms of programmed cell death, providing an analogical basis for the mechanism identified in this study that lncRNA-XIST participates in HF progression via affecting miR-663a/GPX4-mediated ferroptosis. There is bidirectional regulation between RNA and the Wnt/β-catenin pathway: Wnt signaling can affect splicing and stability of RNA, while the mRNAs of key factors in the Wnt pathway are also finely regulated by post-transcriptional modifications, forming a dynamic feedback network (Sun et al., 2025). It is further suggested that in complex pathological processes such as fibrosis, post-transcriptional regulation mediated by non-coding RNAs is deeply intertwined with classical signaling pathways. The lncRNA-XIST/miR-663a/GPX4 axis identified in this study is a concrete manifestation of this regulatory paradigm. To sum up, the aforementioned advances provide solid theoretical support and a broad research perspective for the scientific rationale of this study from multiple perspectives, including common pathways, post-translational modifications, the functional diversity of lncRNAs, and the interaction between RNA and signaling networks.

To our knowledge, this study elucidated the molecular mechanism by which lncRNA-XIST could act as a ceRNA of miR-663a to regulate GPX4 expression to inhibit ferroptosis and promote HSC activation, thereby promoting HF in mice. This regulatory axis not only deepens the understanding of the molecular mechanisms underlying HF but also indicates multi-dimensional potential for clinical translation. From a therapeutic perspective, targeting this ceRNA axis may offer a novel strategy for anti-fibrotic intervention (Riaz and Li, 2019). On the one hand, specific inhibition of lncRNA-XIST expression using siRNA or antisense oligonucleotides could relieve its sponge effect on miR-663a and restore the negative regulation of GPX4 by miR-663a, thereby weakening the survival capability of HSCs and promoting ferroptosis. On the other hand, exogenous delivery of miR-663a mimic could directly enhance its inhibitory effect on GPX4. Regarding diagnosis, lncRNA-XIST and miR-663a have potential as non-invasive biomarkers. Studies have shown that various lncRNAs and miRNAs are stably present in serum or plasma and correlate with liver disease severity (Liu et al., 2022; Li Q. Y. et al., 2023). The present study found that lncRNA-XIST was upregulated and miR-663a was downregulated in fibrotic liver tissues, suggesting their expression imbalance might reflect disease activity. If the correlation between serum lncRNA-XIST/miR-663a ratio and HF staging can be validated in large cohort studies in the future, it holds promise for developing a simple, low-cost liquid biopsy tool to assist in early disease screening or dynamic monitoring of therapeutic response. Furthermore, exploring new strategies, integrating modulators of the lncRNA-XIST/miR-663a/GPX4 axis with existing anti-fibrotic drugs (such as FXR agonists, PPARγ agonists) (Kremoser, 2021; Boyer-Diaz et al., 2021), may contribute to the development of novel and promising therapeutics. Therefore, in-depth exploration of the diagnostic and therapeutic value of the lncRNA-XIST/miR-663a/GPX4 axis may open new avenues for the precise management of HF.

Nonetheless, there are several limitations that need to be considered. First, all functional experiments were only conducted in the immortalized human HSC cell line LX-2, and have not yet been validated in primary HSCs. Although the LX-2 cell line is extensively used in HF research due to its stable activated phenotype and good practicality (Zhou et al., 2025; Yuan et al., 2025), as an immortalized cell line, it may differ from primary HSCs regarding certain biological characteristics. This may affect the physiological relevance of the identified mechanisms and thus weaken the reliability of the conclusions. Therefore, key experiments should be repeated in primary HSCs in the future to strengthen the biological credibility and physiological relevance of the findings. Secondly, the study lacks clinical validation, as expression changes of this regulatory axis have not been examined in liver tissues or serum from HF patients, limiting its translational potential. Subsequent work will involve collecting clinical samples from patients at different fibrosis stages to assess the correlation among lncRNA-XIST, miR-663a, and GPX4 expression and their association with disease progression. Third, the temporal dynamics of this axis during the progression of HF were not investigated, making it difficult to determine whether lncRNA-XIST acts as a driver of fibrosis initiation or represents a secondary adaptive response. This gap affects the judgment of optimal intervention timing. In the future, we will establish time-gradient animal models and combine histopathological scoring with dynamic monitoring of molecular markers to clarify their causal role. Fourth, the implementation of animal experiments only in male mice is a potential sex bias. Existing evidence has indicated that sex significantly influences liver inflammation and fibrotic progression (Wu X. N. et al., 2024; Sayaf et al., 2023). Therefore, female mice should also be enrolled in the animal experiment in future studies to systematically compare expression differences and functional effects of the lncRNA-XIST/miR-663a/GPX4 axis between sexes and evaluate potential sex-dependent characteristics. Furthermore, a recent study highlights the central role of super-enhancer-driven transcriptional regulatory networks in cell fate determination and diseases, including organ fibrosis (Qian et al., 2023). In the present study, lncRNA-XIST was found to be upregulated and functionally critical in HF, and its regulatory function might be under fine control by advanced transcriptional programs. For instance, the SEanalysis 2.0 tool provided by Qian et al. (http://licpathway.net/SEanalysis) enables the analysis of super-enhancer-associated lncRNAs in humans and mice. Application of this tool can help predict whether potential super-enhancers exist near the lncRNA-XIST locus and evaluate their dynamic changes in fibrosis models. Although the present study did not directly validate this upstream regulation, such an analysis would help position lncRNA-XIST within a more complete transcriptional regulatory network. In the future, we will explore whether it acts as a downstream effector molecule of super-enhancer networks to integrate upstream signals and amplify pro-fibrotic transcriptional programs.

## Data Availability

All data generated or analysed during this study are included in this article. Further enquiries can be directed to the corresponding author.
