# Supplementary material for: Long noncoding RNA X-inactive-specific transcript promotes hepatic fibrosis by suppressing ferroptosis in hepatic stellate cells via the miR-663a/GPX4 axis
Source: Front Physiol. 2026 Feb 11;16:1734886. doi: 10.3389/fphys.2025.1734886 (PMC12932219; doi:10.3389/fphys.2025.1734886)
Supplement: Supplementary file 1 [file Supplementaryfile1.docx]

**Supplementary Table 1** Treatment conditions of LX-2 cell group

| Group | Treatment conditions | Biological replicates |
| --- | --- | --- |
| Con | LX-2 cells were cultured in standard condition for 24 h | 3 |
| LX-2 | LX-2 cells were induced with 100 mM ethanol for 24 h | 3 |
| LX-2 + si-NC | 1LX-2 cells were induced with 100 mM ethanol for 24 h and then treated with si-NC (10 nM) for 48 h | 3 |
| LX-2 + si-XIST | LX-2 cells were induced with 100 mM ethanol for 24 h and then treated with si-XIST (10 nM) for 48 h | 3 |
| LX-2 + si-XIST + Vehicle | LX-2 cells were treated with 100 mM ethanol and si-XIST (10 nM) and then treated with an equal volume of solvent for 48 h | 3 |
| LX-2 + si-XIST + Li | LX-2 cells were treated with 100 mM ethanol and si-XIST (10 nM) and then treated with 1 μM Li for 48 h | 3 |
| LX-2 + si-XIST + inhi-NC | LX-2 cells were treated with 100 mM ethanol and 10 nM si-XIST and treated with 10 nM inhibitor NC for 48 h | 3 |
| LX-2 + si-XIST + miR-663a inhi | LX-2 cells were treated with 100 mM ethanol and 10 nM si-XIST, and then treated with 10 nM miR-663a inhibitor for 48 h | 3 |

Note: si, small interfering RNA; NC, negative control; inhi, inhibitor; Li, Liproxstatin-1.
